# Core-Level Photoelectron Angular Distributions at the Liquid–Vapor Interface

**DOI:** 10.1021/acs.accounts.2c00678

**Published:** 2023-01-25

**Authors:** Rémi Dupuy, Stephan Thürmer, Clemens Richter, Tillmann Buttersack, Florian Trinter, Bernd Winter, Hendrik Bluhm

**Affiliations:** † Fritz-Haber-Institut der Max-Planck-Gesellschaft, Faradayweg 4-6, 14195Berlin, Germany; ‡ Department of Chemistry, Graduate School of Science, Kyoto University, Kitashirakawa-Oiwakecho, Sakyo-Ku, Kyoto606-8502, Japan; § Institut für Kernphysik, Goethe-Universität Frankfurt am Main, Max-von-Laue-Strasse 1, Frankfurt am Main60438, Germany

## Abstract

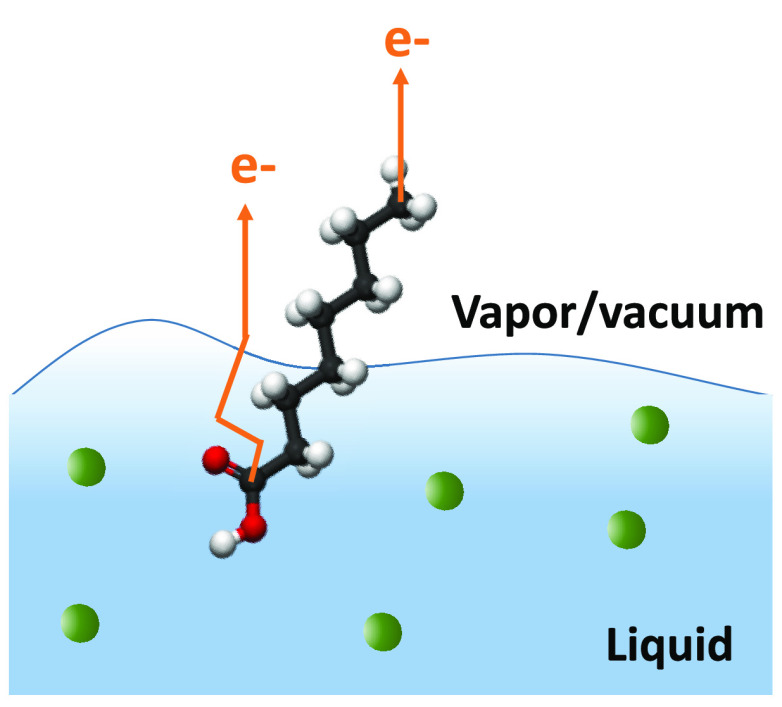

Photoelectron spectroscopy (PES) is a powerful tool for the investigation of liquid–vapor interfaces, with applications in many fields from environmental chemistry to fundamental physics. Among the aspects that have been addressed with PES is the question of how molecules and ions arrange and distribute themselves within the interface, that is, the first few nanometers into solution. This information is of crucial importance, for instance, for atmospheric chemistry, to determine which species are exposed in what concentration to the gas-phase environment. Other topics of interest include the surface propensity of surfactants, their tendency for orientation and self-assembly, as well as ion double layers beneath the liquid–vapor interface. The chemical specificity and surface sensitivity of PES make it in principle well suited for this endeavor. Ideally, one would want to access complete atomic-density distributions along the surface normal, which, however, is difficult to achieve experimentally for reasons to be outlined in this Account. A major complication is the lack of accurate information on electron transport and scattering properties, especially in the kinetic-energy regime below 100 eV, a pre-requisite to retrieving the depth information contained in photoelectron signals.

In this Account, we discuss the measurement of the photoelectron angular distributions (PADs) as a way to obtain depth information. Photoelectrons scatter with a certain probability when moving through the bulk liquid before being expelled into a vacuum. Elastic scattering changes the electron direction without a change in the electron kinetic energy, in contrast to inelastic scattering. Random elastic-scattering events usually lead to a reduction of the measured anisotropy as compared to the initial, that is, nascent PAD. This effect that would be considered parasitic when attempting to retrieve information on photoionization dynamics from nascent liquid-phase PADs can be turned into a powerful tool to access information on elastic scattering, and hence probing depth, by measuring core-level PADs. Core-level PADs are relatively unaffected by effects other than elastic scattering, such as orbital character changes due to solvation. By comparing a molecule’s gas-phase angular anisotropy, assumed to represent the nascent PAD, with its liquid-phase anisotropy, one can estimate the magnitude of elastic versus inelastic scattering experienced by photoelectrons on their way to the surface from the site at which they were generated. Scattering events increase with increasing depth into solution, and thus it is possible to correlate the observed reduction in angular anisotropy with the depth below the surface along the surface normal.

We will showcase this approach for a few examples. In particular, our recent works on surfactant molecules demonstrated that one can indeed probe atomic distances within these molecules with a high sensitivity of ∼1 Å resolution along the surface normal. We were also able to show that the anisotropy reduction scales linearly with the distance along the surface normal within certain limits. The limits and prospects of this technique are discussed at the end, with a focus on possible future applications, including depth profiling at solid–vapor interfaces.

## Key References

- Thürmer, S.; Seidel, R.; Faubel, M.; Eberhardt, W.; Hemminger, J. C.; Bradforth, S. E.; Winter, B. Photoelectron Angular Distributions from Liquid Water: Effects of Electron Scattering. Phys. Rev. Lett.2013, 111, 1730052420648710.1103/PhysRevLett.111.173005.^1^ This seminal study reported the first measured photoelectron angular distributions (PADs) from the O 1s core level of neat liquid water. The idea of accessing scattering parameters through the measurement of PADs was introduced here.
- Dupuy, R.; Filser, J.; Richter, C.; Seidel, R.; Trinter, F.; Buttersack, T.; Nicolas, C.; Bozek, J.; Hergenhahn, U.; Oberhofer, H.; Winter, B.; Reuter, K.; Bluhm, H. Photoelectron angular distributions as sensitive probes of surfactant layer structure at the liquid–vapor interface. Phys. Chem. Chem. Phys.2022, 24, 4796–48083515666810.1039/d1cp05621bPMC8865751.^2^ This study on PADs from surfactant solutions demonstrated the capability of the technique to study the relative depth of different molecules at the liquid interface, but also the surface arrangement of a given molecule through probing the average depth of functional groups within that molecule.
- Dupuy, R.; Filser, J.; Richter, C.; Buttersack, T.; Trinter, F.; Gholami, S.; Seidel, R.; Nicolas, C.; Bozek, J.; Egger, D.; Oberhofer, H.; Thürmer, S.; Hergenhahn, U.; Reuter, K.; Winter, B.; Bluhm, H. Angstrom depth resolution with chemical specificity at the liquid−vapor interface. arXiv:2209.1543710.1103/PhysRevLett.130.15690137115858.^3^ Here, we used PAD measurements of perfluorinated surfactants with four distinguishable carbon atoms to demonstrate that the anisotropy parameter of the PAD correlates linearly with the average depth of the probed site in the surfactant molecule. The achievable depth resolution was estimated to be close to 1 Å.

## 1 Introduction

Photoemission spectroscopy (PES) has been applied to high-vapor-pressure liquids, most importantly water, for more than 20 years now, due to the parallel developments of liquid-microjet photoemission spectroscopy (LJ-PES)^4^ and ambient pressure X-ray photoelectron spectroscopy (APXPS).^5^ Many tools and techniques based on PES, developed initially in the context of solid-phase, ultra-high-vacuum surface science, have been transferred to the investigation of liquid interfaces with great success.^6−9^ One important application for both liquid and solid interfaces is the determination of depth profiles, that is, the distribution of species as a function of depth into bulk. The most common method to obtain this information is to vary the depth sensitivity of the measurement, characterized by the effective attenuation length (EAL), the mean distance until the PE signal attenuates to 1/*e*, which depends on the take-off angle relative to the surface normal and on the kinetic energy of the electrons (eKE). The latter determines the mean free paths of both elastic (EMFP) and inelastic scattering (IMFP), that is, the mean path length until the electron encounters a scattering event. Inelastic scattering effectively removes photoelectrons from the relevant signal (see details in, e.g., refs (6) and (7)). Changing the take-off angle to obtain depth information has been done before on liquid interfaces^10,11^ but is not suited for non-flat geometries, such as the overwhelmingly used cylindrical microjet geometry. Changing the probability for scattering, that is, the MFPs, however, can be done by varying the photon energy, which in turn changes the eKE. This has been largely exploited in early PES studies on liquids (see, e.g., ref (12)).

There are nonetheless several challenges when applying kinetic-energy depth profiling to liquids.^3,6^ One important issue is the lack of understanding of electron transport and scattering parameters, as well as photoionization cross sections.^13^ Another challenge is the fluctuating nature of the liquid surface itself, where simplifying assumptions have to be made during analysis. This is less of an issue in the solid phase, where it is possible to grow layers of known thickness and composition, which enables a precise determination of the electron-scattering parameters, and to test analysis procedures on well-controlled structures. Research carried out over many decades on electron transport and surface characterization for solid samples is thus available. This is not the case for liquid surfaces, and thus scattering parameters for water await further experimental studies and current conclusions are under debate.^14−17^ In addition, while ambiguities in the solid phase can also be circumvented using other advanced PES techniques, such as X-ray standing wave spectroscopy^18^ or peak-shape analysis,^19^ these approaches cannot be applied for the investigation of liquid–vapor interfaces. Recently, we have described a novel method to investigate the interfacial depth structure in liquids via the measurement of core-level photoelectron angular distributions (PADs).^2,3,20^

PADs of isolated molecules characterize the interplay between the outgoing photoelectron and the molecular potential, yielding information on the latter, as well as on orbital character and photoionization dynamics.^21^ For randomly oriented molecules in the gas phase interacting with linearly polarized light, the PAD is described by the following equation:^22,23^
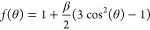
1where θ is the emission angle relative to the linear polarization direction and β is the anisotropy parameter that describes the distribution. At the so-called magic angle (54.7°), where photoemission becomes independent of β, eq 1 reduces to 1. Interaction with unpolarized^6^ or circularly polarized^24−26^ light gives rise to other types of anisotropies,^21^ which will not be discussed here. For closed-shell atomic, fully symmetric s orbitals, β = 2 for all eKEs, while in a more general case β takes values between −1 and 2 (with β = 0 corresponding to a fully isotropic distribution) and depends on the eKE. In molecules, β is further affected by interaction with the molecular potential, which can be interpreted as intramolecular scattering of the outgoing photoelectron wave.

Analogously, β also reflects changes in the orbital character in isotropic, amorphous condensed-phase systems, that is, where eq 1 applies (the case of oriented systems is briefly discussed in section 4). Information on the effects of condensation on molecular orbitals, such as changes in orbital shapes, can in principle be obtained from PADs, and this has been attempted for the valence orbitals of liquid water.^27−29^ The study of entities specific to the condensed phase such as the solvated electron is also possible,^30^ for example, in water clusters^31^ or liquid water.^32^

A major hurdle, however, is the inevitable modification of experimental PADs by elastic scattering in the condensed phase. Elastic scattering changes the electron trajectories and thus modifies the observed angular distributions, usually leading to a reduction of the measured anisotropy. To access the true, or “nascent”, PAD that reflects photoionization dynamics, one needs to account for this process, which is in fact a major contribution to the measured PADs in liquids.^1,27^ Sophisticated electron-transport models are required and have been developed to retrieve nascent distributions from measured PADs, as described in a recent review.^30^ The retrieval of nascent PADs is mostly relevant for valence orbitals. Core levels, however, primarily have atomic character and are thus not expected to be significantly altered as compared to isolated systems. This means core-level PADs in the amorphous condensed phase exclusively inform on electron scattering. Scattering is thus no longer a parasitic effect obscuring the nascent distribution, but is exactly the property we want to measure.

Besides enabling quantification of electron-scattering processes in the condensed phase (key ref (1), see also ref (30)), we have shown recently that PADs can be used to perform chemically sensitive depth profiling of the liquid–vacuum interface (key references (2) and (3), see also ref (20)), exploiting the difference of elastic scattering experienced by photoelectrons emitted by atoms located at different distances from the surface. These two topics will be tackled after a brief discussion of the experimental requirements for these measurements as well as a few theoretical considerations. We close with a discussion of the strengths and limitations of this technique and possible expansion to other interfaces.

## 2 Experimental Considerations

For the gas phase, imaging techniques based on velocity map imaging (VMI)^33^ are often used to directly map PADs over a solid angle of 360°, and the most sophisticated of these measurements, COLTRIMS (cold target recoil ion momentum spectroscopy), allows molecular-frame PAD measurements.^34,35^ The development of VMI combined with a liquid–vapor interface system (i.e., operating necessarily at pressures often exceeding 10^–4^ mbar), such as a liquid microjet, has recently been attempted^36,37^ but remains a technological hurdle.

**Figure 1:**
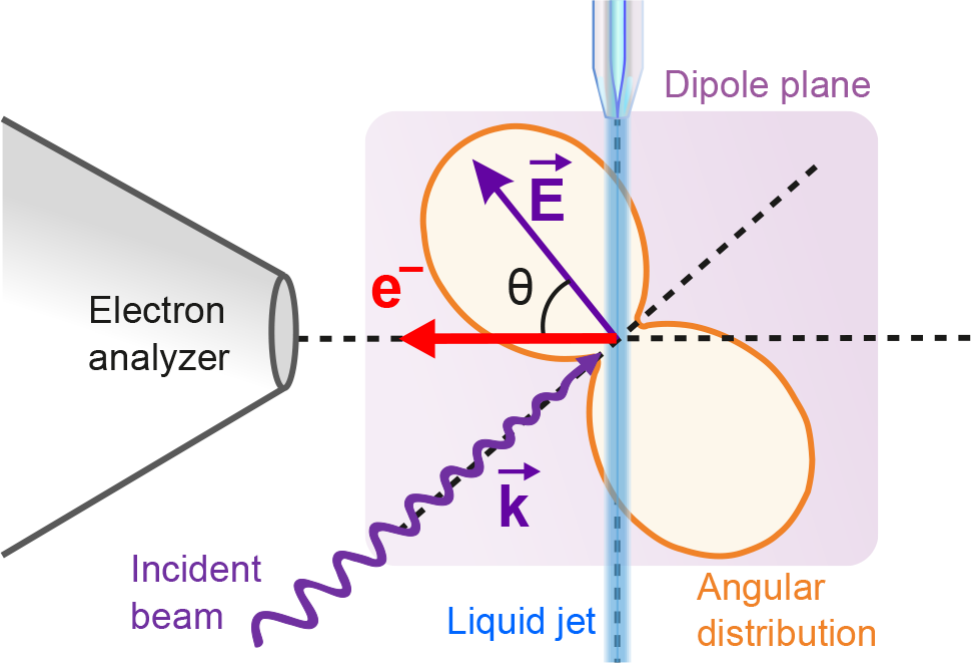
Sketch of the experimental geometry. Light propagation vector k⃗, detection direction, and liquid-jet propagation direction are all orthogonal; that is, the latter two comprise the dipole plane, defined as orthogonal to the light propagation direction. The light polarization vector E⃗ is varied, effectively rotating the angular distribution with respect to the detection direction. Nozzle diameters of 20–40 μm, and jet temperatures between 10 and 25 °C are typically used. The chamber pressure is kept around 10^–3^–10^–4^ mbar.

So far, experiments use a conventional hemispherical electron analyzer (HEA) placed in the dipole plane (i.e., orthogonal to the light propagation direction), and photoelectron spectra are measured at different angles of the linear light polarization vector with respect to the detection direction; see sketch in Figure 1. PE intensity variations as a function of this angle yield the respective PADs *f*(θ), from which the asymmetry parameter β is determined using eq 1.

This method requires the availability of an X-ray source with tunable linear polarization, as is commonly provided by synchrotron-radiation beamlines equipped with an elliptical polarization undulator (EPU). The experiments presented here, for instance, have been performed at the UE52_SGM beamline at the BESSY II synchrotron radiation facility and at the PLEIADES beamline at the SOLEIL synchrotron radiation facility. Further considerations for accurate PAD measurements are discussed in the Supporting Information, including jet stability, photon flux monitoring, alignment, and other experimental factors. We introduce a modified expression of eq 1 for a practical analysis.

## 3 Results for Neat Water and Theoretical Considerations

**Figure 2:**
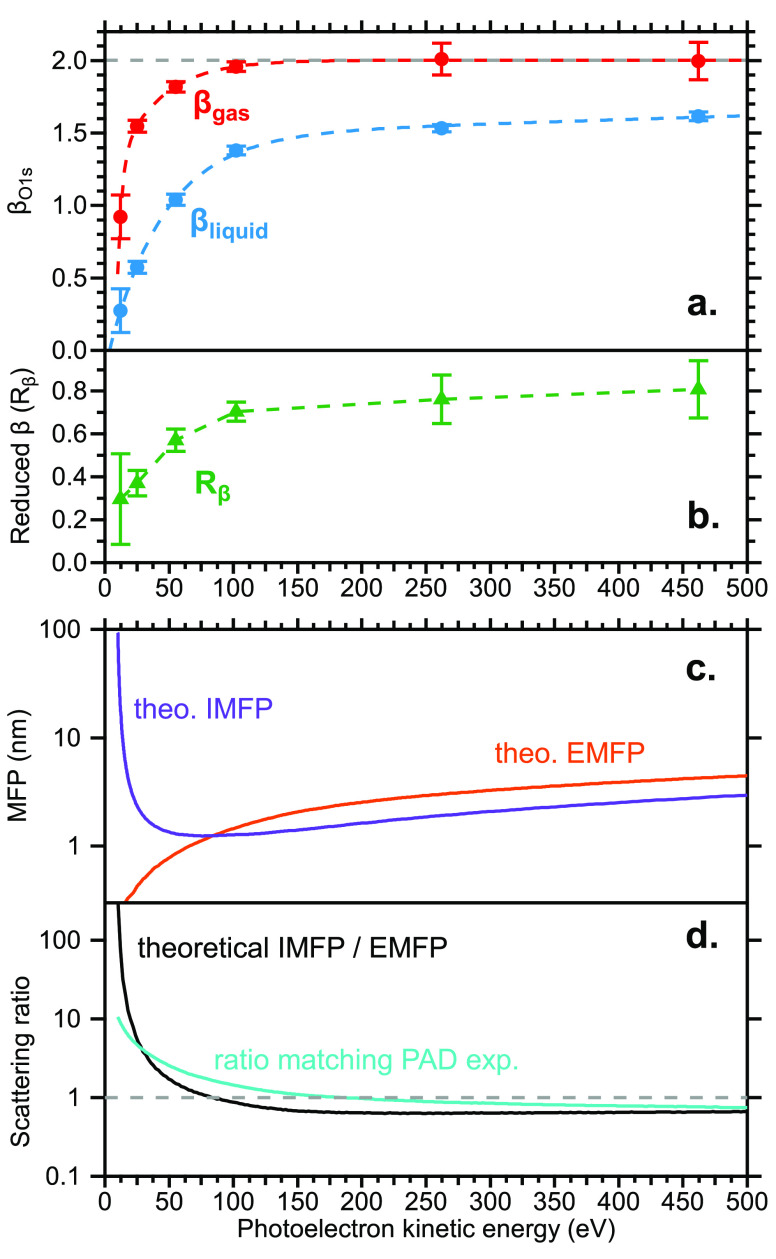
(a) Anisotropy parameter β of O 1s photoelectrons from gaseous (red) and liquid (blue) water as a function of eKE (bottom axis). (b) Reduction of β when going from gas to liquid. (c) Exemplary recent theoretical IMFP (purple) and EMFP (orange) values for liquid water taken from ref (38). (d) Scattering ratio, IMFP/EMFP, indicating the average number of elastic collisions encountered by an electron escaping the surface. The black curve shows the ratio for the curves from panel (c), while the turquoise curve is modeled to match the observed trend in panel (b). Data of panels (a), (b), and the turquoise curve in panel (d) are from ref (1).

The first liquid-phase PADs from a core level have been measured from neat water by Thürmer et al.^1^ and will serve as a basis to discuss some fundamental aspects of PADs in the liquid phase. In this work, the β parameter of gas- and liquid-phase water was measured simultaneously for different eKEs; the results are reproduced in Figure 2a. The value of β for liquid-phase water is observed to be systematically lower than the gas-phase value, from about 20% at high eKE to 60–70% at low eKE. This relative decrease is expressed by the reduced β parameter, defined as *R*_β_ = β_liq_/β_gas_ and shown in panel (b). (Note that this definition is different in the original paper.)

As mentioned in the Introduction and demonstrated in Figure 2, the PAD anisotropy in the condensed phase is reduced by elastic electron scattering. Inelastic scattering, leading to an energy loss of several eV, effectively removes the photoelectron from the signal. Thus, detected photoelectrons have traveled on average a distance equal to the IMFP before escaping the surface. Over such a distance, they have on average encountered a number *n* of elastic scattering events equal to the IMFP/EMFP ratio, which is therefore the determining quantity for anisotropy reduction. This is exemplified by recent theoretical MFPs for liquid water shown in Figure 2c, here, taken from ref (38).

Note that here we consider only a two-channel model of electron scattering, characterized by the IMFP and EMFP. The IMFP is determined by electronic inelastic scattering, which incurs a loss of about 8 or more eV. The EMFP includes both truly elastic scattering with no energy loss, as well as rovibrational scattering channels, which incur energy losses of a few hundreds of millielectronvolts at most. The latter channels are negligible at high eKEs, but can cause peak deformations and shifts, especially at a very low eKE < 15 eV (see refs (30) and (39)). While the EMFP increases monotonically toward higher eKE, the IMFP exhibits a distinct minimum at about 100 eV; an increase toward lower eKE is mainly due to the closing of available electronic inelastic scattering channels. Above 100 eV, the EMFP and IMFP are similar; that is, elastic and inelastic scattering are equally likely. At lower eKE, however, an electron encounters many more elastic-scattering events (due to a much lower EMFP) before being detected [see Figure 2d], thus decreasing the anisotropy. To reproduce the observed experimental *R*_β_ [Figure 2b], a scattering ratio like the turquoise curve in panel (d) is expected. Theory comes close to this expectation, but somewhat overestimates the ratio at very low eKE. This may indicate so far unaccounted scattering channels, which could reduce the IMFP. We also experimentally observed unusually high inelastic scattering at low eKE in a recent study.^39^

To go beyond this qualitative description, we must, however, understand more precisely how elastic scattering modifies the nascent angular distribution. Another key factor is the differential elastic-scattering cross section (DCS). The DCS represents the probability that a photoelectron is scattered in a given direction upon an elastic-scattering event. If *n* is the average number of elastic collisions of the photoelectron, the measured PAD I*(θ) is the *n*-fold convolution of the nascent PAD *I*(θ) by the DCS:^1^
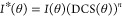
2where the power of *n* implies an *n*-time convolution product.

The DCS depends on the eKE, and its dependence on θ cannot be easily described by an analytical formula. However, in a first approximation, it is possible to gain insight from an analytical description, as was done in key ref (1), where the DCS was approximated by a simple Gaussian function. From eq 2, it is thus possible to derive the average number of elastic collisions *n* (i.e., the IMFP/EMFP ratio) from the experimental data. The result was then used to correct the estimate of the EAL for liquid water previously made by Ottosson et al.^16^ for attenuation effects due to the angular asymmetry. It was also shown that the IMFP/EMFP ratio at low eKE was severely overestimated by theory so far, highlighting our insufficient knowledge of scattering processes in this KE range. As seen in Figure 2d, recent theory results are getting closer, but still tend to overestimate the ratio below 15–20 eV.

A better way to model electron scattering than this simple analytical approach is to use Monte Carlo numerical simulations, that is, probabilistic models that calculate random trajectories for electrons in a given geometry, taking into account elastic and inelastic scattering cross sections (including DCS), to simulate electron spectra and PADs. One example is the free software Simulation of Electron Spectra for Surface Analysis (SESSA) provided by NIST.^40^ SESSA was used previously to simulate electron transport in aqueous solutions^41^ and can also be used to explore PADs, for instance, to gauge how the β parameter responds to changes of a given parameter with all others fixed. An example is given in the Supporting Information. More sophisticated models, dedicated specifically to electron-transport simulations in aqueous systems (liquid water, clusters, droplets, etc.), continue to be improved. Specifically, the angular-distribution data of key ref (1), along with other data sets, have been re-analyzed to yield electron scattering parameters and compare them with other available theoretical data^42^ and ice-phase data.^14^ Conversely, if accurate scattering parameters were available, one can retrieve genuine electron spectra and PADs at low eKEs from experimentally measured ones using these models, as described in detail in ref (30).

As mentioned in the Introduction, we measure core-level PADs in the condensed phase with the intention to access the reduction of anisotropy caused by elastic scattering. These liquid-phase PADs therefore need to be compared to the nascent PADs, which are not known a priori. Our approach here will be to consider that the nascent PAD can be approximated by the measured PAD of the gas-phase species of interest. This approximation is already implicit in the results of key ref (1) outlined above, where we introduced the reduced value *R*_β_ as β_liq_/β_gas_. There is no a priori justification for assuming that the measured β should vary linearly with the nascent β for fixed scattering parameters, but SESSA simulations show it is in fact the case, as shown in the Supporting Information.

One can also question whether gas-phase PADs truly represent the nascent PAD of the molecule in the liquid phase. In the condensed phase, there can be changes in orbital character due to solvation and hydrogen bonding.^29^ We assume this effect can be neglected for core levels, which are almost exclusively atomic in character and only slightly perturbed by condensation. Another possible effect is a change of conformation of the molecule, which would potentially affect intramolecular scattering. It is difficult to evaluate the magnitude of such an effect, which we will therefore also neglect and consider as an additional uncertainty of our results.

## 4 PADs as a Depth-Profiling Technique

**Figure 3:**
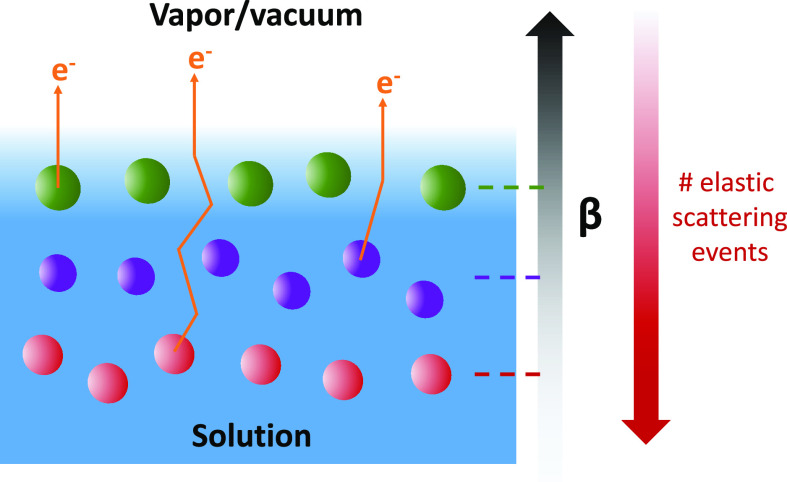
Schematic principle of depth profiling with PADs.

We now turn to the central idea presented in our recent works and outlined in the key references: the use of core-level PADs, containing information on the amount of elastic electron scattering, as a depth-profiling technique. The principle of this idea is sketched in Figure 3: we consider a fictive solute where different (PES-distinguishable) atoms are located at different average positions from the liquid–vacuum interface. Photoelectrons emitted from the atoms located deepest inside the interface will encounter on average more elastic-scattering events than those emitted from the atoms closest to the interface. More elastic-scattering events will lead to a more isotropic PAD, and thus a lower β parameter. In other words, it is in principle possible to use the β parameter as a measure of the average depth of the atoms inside the interface, that is, the distance from the top surface into solution, as will be reviewed below.

In a first study using this approach, Lewis et al.^20^ measured the S 2p PADs of an equimolar mix of organosulfur compounds (DMSO/DMSO_2_) in aqueous solution. They found very similar β values for the two compounds, despite the respective PE signal intensity being overall much higher for DMSO_2_. In the framework developed above, the similar β values for the two species indicate similar scattering behavior of the photoelectrons, and thus similar average depth distributions of the two molecules. The higher PE signal intensity for DMSO_2_ can then unambiguously be attributed to a higher surface density of this compound, despite their equal concentration in the bulk solution.

These results demonstrate that PADs can help resolve one fundamental ambiguity that exists in quantitative interpretation of PE signals: the entanglement of surface density and depth profiles. It has been recognized since the beginning of quantitative analysis of PES data that signal intensities are intrinsically ambiguous^19^ because higher/lower atomic densities and deeper/shallower depth distributions can give rise to similar PE intensities, and vice versa. This fundamental issue has been addressed in different ways, as reviewed in the Introduction, which work relatively well in the case of solid interfaces, but have remained problematic for liquid interfaces before the introduction of the PAD technique.

In a recent study, we performed PAD experiments on a medium-sized surfactant molecule, octanoic acid, and its deprotonated counterpart, (sodium) octanoate.^2^ These molecules have two PE-distinguishable carbon atoms: the functional COOH/COO^–^ carbon and the CH_*x*_ carbons of the aliphatic chain. It is therefore possible to investigate whether the PADs of these two sites of the same molecule differ. That study also stressed the need to perform measurements from the respective gas-phase molecule, as β_gas_ already differs for different functional groups, even within the same atomic shell (C 1s in this case). Indeed, for gas-phase pentanoic acid (a proxy for the less volatile octanoic acid) and at eKE ∼150 eV, we found β(CH_*x*_) = 1.96 ± 0.03 and β(COOH) = 1.87 ± 0.03, a significant difference.

**Figure 4:**
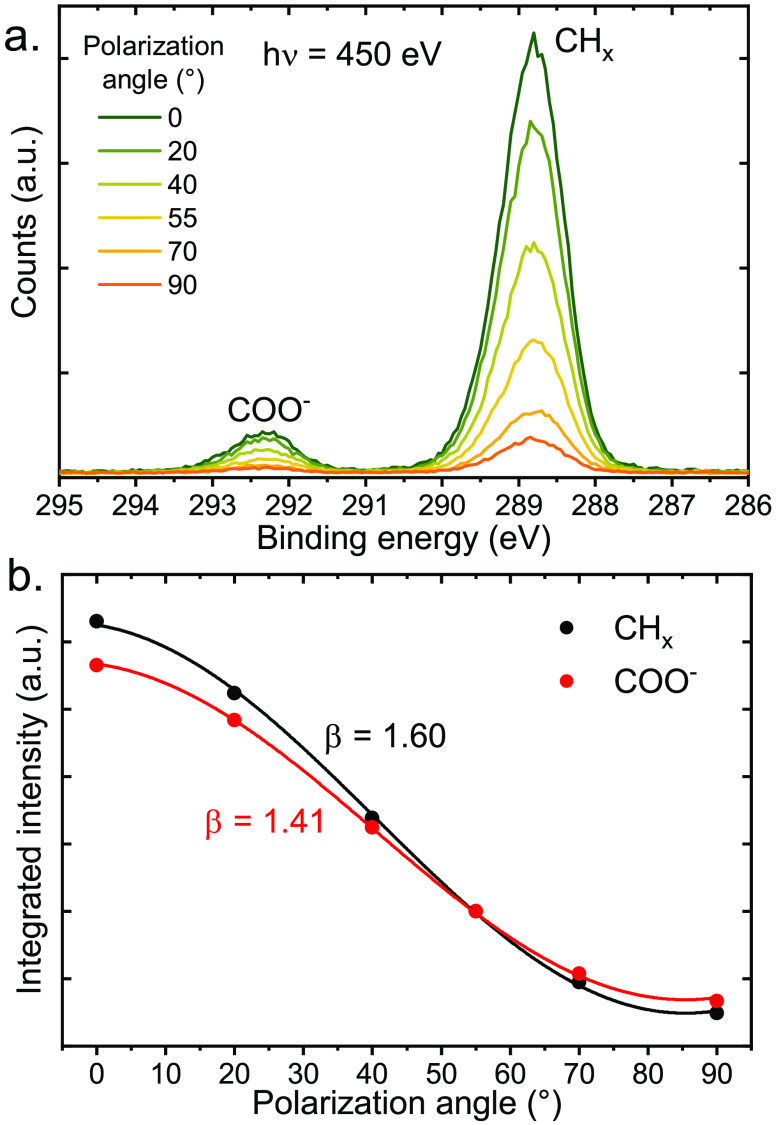
PADs of the C 1s levels (450 eV photon energy, i.e., eKE ∼150 eV) of a 100 mM sodium octanoate solution. (a) C 1s PE spectra, where the COO^–^ and CH_*x*_ carbons are identified by a large chemical shift of 3.5 eV. Spectra were measured at a few distinct polarization angles, resulting in intensity variations of the peaks. (b) Integrated intensities of the two peaks from panel (a) as a function of the polarization angle. The data were fitted with eq S1 (*p* = 1) to extract the β parameters. Reproduced with permission from ref (2). Copyright 2022 Royal Society of Chemistry.

Even after normalization by the gas-phase values, we found differences, attributed to different amounts of elastic scattering, between the anisotropies of the CH_*x*_ and COOH (COO^–^, respectively) groups for octanoic acid (sodium octanoate, respectively) solutions. The difference is significant for sodium octanoate, where for a 100 mM solution *R*_β_(CH_*x*_) = 0.82 ± 0.02 and *R*_β_(COO^–^) = 0.76 ± 0.02 (note that in key ref (2) a different normalization convention was used). The spectra and PADs for this particular solution are shown in Figure 4. This clearly indicates that the COO^–^ group, as the hydrophilic anchor of the molecule, is located on average deeper into solution than the aliphatic chain carbons. In contrast, for a 4 mM octanoic-acid solution, *R*_β_ values are almost identical (*R*_β_(CH_*x*_) = 0.84 ± 0.02 and *R*_β_(COOH) = 0.83 ± 0.02), indicative of an almost flat arrangement of the molecule in the surface plane (i.e., an almost equal average depth for all carbons). These conclusions were confirmed by molecular dynamics (MD) simulations. This underscores the capability of core-level PADs to reveal the arrangement of a given molecule at the interface, by probing the average depth of its different functional groups.

**Figure 5:**
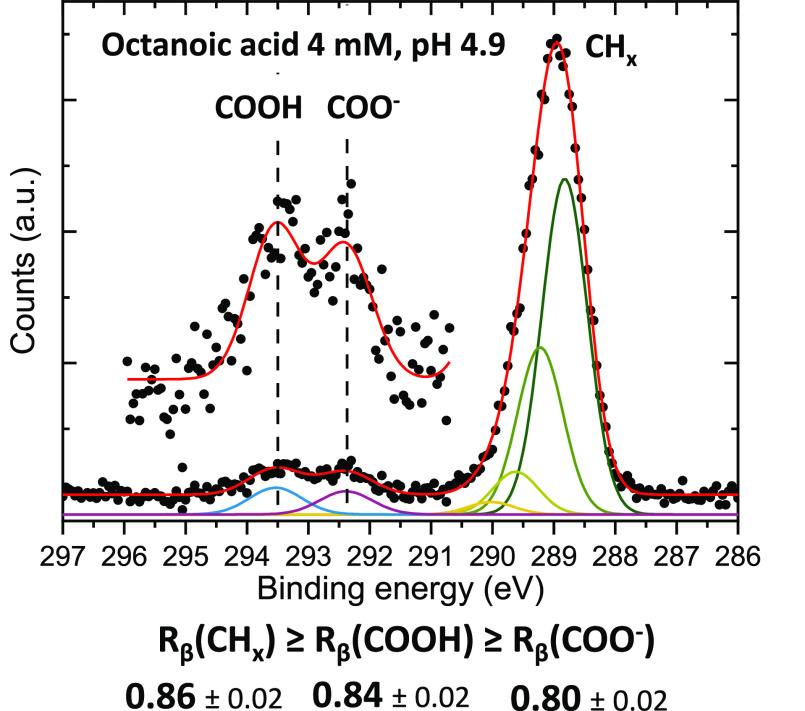
C 1s PE spectrum of a 4 mM octanoic-acid solution at pH 4.9, where both species (octanoic acid and octanoate) are present in equal proportion at the surface, measured at the magic angle (54.7°) and with 450 eV photon energy. The protonated and deprotonated species are well separated. Normalized *R*_β_ parameters for the three peaks are given below. Data were adapted from ref (2).

As explained in ref (2), comparison of *R*_β_ values from different solutions is difficult, because they do not necessarily have the same scattering properties (especially if, e.g., the surface density of surfactants is different). We were thus not able to directly compare octanoic-acid and octanoate solutions. However, we could measure both species from a solution with a pH close to the p*K*_a_ value (∼4.9). A single PE spectrum from this solution, shown in Figure 5 and measured at magic angle (54.7°), does not reveal information on the orientation, surface density, and relative depth of the molecules at the interface, because all of these properties are entangled. However, measurement of the *R*_β_ parameters, indicated in Figure 5, yields a clear hierarchical order of the relative depth of the different probed functional groups, showing, for instance, that the COO^–^ carbons are located deeper in the interface than are the COOH carbons. We are thus able to infer the relative depth of different species, with different surface propensities, in a more complex (i.e., not single-solute) solution. This is again confirmed by MD simulations.

**Figure 6:**
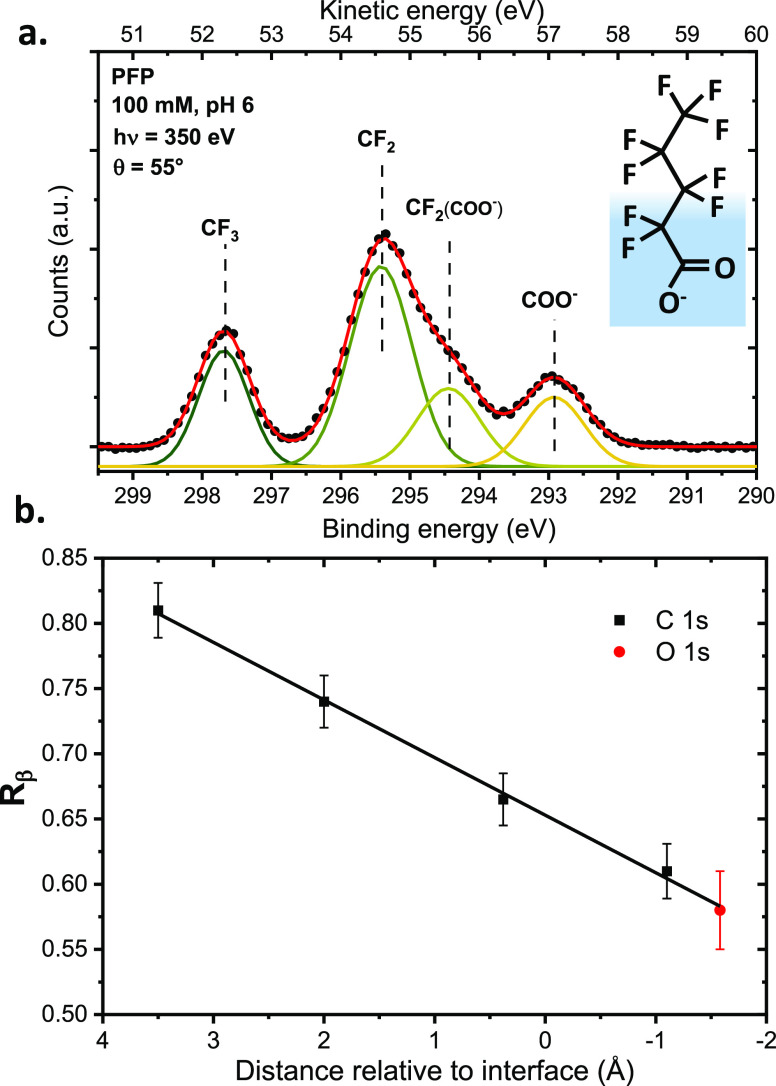
(a) C 1s XPS spectrum of a 100 mM NaPFP aqueous solution at pH 6, measured at a photon energy of 350 eV and at the magic angle (54.7°). The PFP molecule is sketched to the left, with its presumed orientation relative to the liquid–vacuum interface. Four peaks can be distinguished and are attributed in the figure. (b) Extracted *R*_β_ values for all four carbon peaks as well as for the O 1s peak of the COO^–^ oxygen atoms, plotted against their distance to the water interface, as determined from MD simulations. Data were adapted from ref (3).

Further developing the technique, in key ref (3) we explored the depth resolution limits that can be achieved.^3^ For this purpose, we chose a surfactant molecule where it is possible to distinguish as many sites as possible in the PE spectrum: perfluorinated pentanoic acid (PFPA), or rather its deprotonated counterpart, sodium perfluoropentanoate (PFP). As shown in Figure 6a, the C 1s PE spectrum of PFP in aqueous solution exhibits four distinct carbon peaks, out of five carbons in the molecule. This allows us to probe selectively along the molecular chain. Like octanoate, PFP is expected to orient itself straight along the surface normal, which was confirmed by MD simulations. It is thus possible, by measuring PADs of the four distinct carbons, to relate their *R*_β_ value to their well-defined distance within the interface along the surface normal. This is presented in Figure 6b, where we also show *R*_β_ measured for the COO^–^ oxygen atoms (O 1s level). *R*_β_ values, extracted at eKE ∼50 eV, are plotted against the average distance to the interface as determined from MD simulations.

Three important conclusions can be drawn from these data. First, one observes a linear relationship between *R*_β_ and the distance relative from the interface. Using the analytical approximation developed in section 2, it can be shown that such a linear relationship is expected for a low number of elastic collisions (*n* → 0, in practice *n* of the order of 1), as shown in the Supporting Information (eq S5). The definition for this low-collision regime will depend on the scattering properties of the system, that is, the values of the EMFP, IMFP, and the DCS. For an estimated EMFP of the order of 5–8 Å at eKE = 50 eV,^1^ and with a length of ∼6 Å for the molecule, linearity is indeed expected within the PFP layer itself.

Furthermore, the linear *R*_β_ scale extends across measurements from different atomic species, as seen from the alignment of the O 1s data point with the C 1s points in Figure 6b. Finally, the spatial sensitivity achieved is excellent: carbon atoms separated by about 1.5 Å can be distinguished, implying that even sub-Å resolution is possible, depending on the achievable error bars (of the order of 0.04 on the *R*_β_ scale) and the slope of the data in Figure 6b (0.045 per Å). We could also show in ref (3), by measuring the analogous data for different eKEs, that the slope (and, thus, the sensitivity) depends on eKE, following the expected behavior of a larger slope at lower eKE where the EMFP is shorter.

## 5 Conclusion and Outlook

The examples detailed in this Account show how core-level PADs in the condensed (liquid) phase reveal elastic scattering and how this can be exploited to gain depth information. Quantitative parameters on scattering can be retrieved, which is of great use for modeling electron transport. We found that core-level PADs can be utilized for depth profiling with excellent element and spatial sensitivity and chemical specificity, which we demonstrated for solutions of surfactant molecules. An interesting expansion of the technique may be the application to other basic systems, for example, inorganic ion pairs (Na^+^, K^+^, Cl^–^, I^–^, etc.) in aqueous solution, whose propensity for the interface has been actively studied in the field a decade ago.^43−45^ Future studies would need to determine whether it is possible to establish (for exemplary systems or even in general) an absolute *R*_β_ scale by experimental means. Indeed, the establishment of an *R*_β_ scale in key ref (3), its linearity, and the sensitivity estimations have all relied on MD simulations to provide an absolute scale in terms of atomic distribution along the surface normal. Otherwise, the information would remain essentially qualitative.

How does the PAD method compare to other techniques of obtaining depth information at the molecular scale? For liquid surfaces, aside from PES, mainly two methods have been applied: X-ray reflectivity (XRR)^46,47^ and, less commonly, neutral backscattering.^48^ Both can achieve depth profiles with sensitivity down to 2–3 Å, and some degree of elemental specificity: XRR by tuning to a resonance and neutral backscattering by a complex analysis of the data. This elemental specificity, however, does not equate with the chemical specificity (in the sense of distinguishing the chemical state of various elements) achieved in PES-based techniques. In that sense, PAD depth profiling offers complementary information to these techniques. We also mentioned throughout the text the limits of other photoemission-based techniques used to retrieve depth profiles. PAD-based depth profiling therefore brings additional capabilities to the photoemission toolbox.

All works on core-level PADs in the liquid phase so far have been performed on cylindrical microjets. Equation 1 is only valid for randomly oriented molecules, and the cylindrical geometry, while not strictly yielding a random orientation of molecules, introduces sufficient averaging over multiple orientations, electron take-off angles, and electron analyzer acceptance angles so that, in conjunction with the disorder inherent to the liquid surface, the random approximation is reasonable. So far, core-level PADs never showed any higher-order cos(θ) dependence of the PAD. This could potentially be different in a flat-surface geometry, where molecular orientation could translate into more complex PADs, but such measurements have not been reported so far. In this regard, the recently developed flat jets for PES,^49^ the flat equivalent of regular cylindrical microjets, represent an exciting development. Another possibility may be to use static or flowing liquid geometries under thermodynamic equilibrium, that is, at water vapor pressures up to 10–20 mbar. Photoemission on, for example, Langmuir troughs, rotating disks,^50^ or liquid lamellas^10^ has been demonstrated, but the equilibrium-pressure conditions might hamper PAD measurements because of elevated electron scattering in the gas phase.

While all studies presented here have been performed on liquid-phase systems, analogous PAD measurements from the solid interface are feasible. The possibility to extract scattering information from PADs has been recognized previously in the case of free nanoparticles,^51^ but not applied to depth profiling as far as we know. For amorphous, solid disordered systems, for example, amorphous films, core-level PADs could similarly reveal precious depth information.

## Supporting Information Available

Additional experimental details and a description of scattering simulations using SESSA (PDF)
