# Supplementary material for: Core-Level Photoelectron Angular Distributions at the Liquid–Vapor Interface
Source: Acc Chem Res. 2023 Jan 25;56(3):215–23. doi: 10.1021/acs.accounts.2c00678 (PMC9910046; doi:10.1021/acs.accounts.2c00678)
Supplement: Supplementary file 1 — ar2c00678_si_001.pdf [file ar2c00678_si_001.pdf]

Supporting Information

Core-level photoelectron angular distributions at the liquid-vapor interface

Rémi Dupuy[1,*], Stephan Thürmer[2], Clemens Richter[1], Tillmann Buttersack[1], Florian Trinter[1,3], Bernd Winter[1] and Hendrik Bluhm[1,*]

[1] Fritz-Haber-Institut der Max-Planck-Gesellschaft, Faradayweg 4-6, 14195 Berlin, Germany
[2] Department of Chemistry, Graduate School of Science, Kyoto University, Kitashirakawa-Oiwakecho, Sakyo-Ku, Kyoto 606-8502, Japan
[3] Institut für Kernphysik, Goethe-Universität Frankfurt am Main, Max-von-Laue-Str. 1, 60438 Frankfurt am Main, Germany

1 Experimental Details

The measurements rely on comparing absolute photoelectron intensities for successively measured spectra at different polarization angles. This requires particularly stable conditions. Photon-flux variations (including differences between different polarization angles) need to be measured reliably. This is typically done using a calibrated photodiode ahead of the experimental chamber, or by monitoring the mirror current of the last mirror of the beamline. Typically, drifts in the intensity due to an unstable liquid-jet or beam-focus position are monitored by regularly re-measuring at one reference polarization angle (usually 0° as it is the most intense one). The measurements are also affected by the necessarily non-perfect measurement conditions. To discuss the various sources of imperfections, we first introduce a modified version of Eq. 1 from the main text that is effectively used for fitting data:

$$f(\theta) = 1 + \frac{\beta}{4}(1 + 3p\cos(2(\theta - \theta_0))) \tag{S1}$$

Here, θ_0 is an offset of the polarization angle and p is the degree of linear polarization (Stokes parameter) of the light. This equation is equivalent to Eq. 1 of the main text for p = 1 and $\theta_0 = 0$. An offset θ_0 of the polarization angle can stem from an actual offset of the true polarization direction relative to the nominal one, originating, *e.g.*, from a small mechanical offset of the undulator, from to slight deviations from the experimental geometry sketched in the main text (i.e., alignment of the detector relative to the light propagation axis), or even possibly stray magnetic fields in the chamber that bend electron trajectories. θ_0 can be determined from the fit. The degree of linear polarization of the light p, on the other hand, stems from a non-ideal linear (*i.e.*, slightly elliptic) polarization, but cannot be determined from fitting because the p parameter is quasi-degenerate with the β parameter. On the other hand, that means that if p is different from 1, this will result in an overall reduction of the fitted β values compared with the true ones. If one only wishes to compare relative β values, which is our case here, this is of little importance.

Another factor is the non-zero angular acceptance of the detector, meaning a measurement at a certain angle actually averages over an angular range. This effectively lowers all the measured values of β uniformly, just like depolarization effects. One can theoretically calculate what would be the effect of a non-zero circular angular acceptance on an initial PAD with anisotropy parameter β. The modified distribution reads:

$$f^*(\theta) = 1 + \frac{\sin(2\theta_a)}{2\theta_a}\frac{\beta}{2}\left(3\cos^2(\theta) - \frac{3}{2} + \frac{\theta_a}{\sin(2\theta_a)}\right) \tag{S2}$$

where θ_a is the half-acceptance angle. Thus, for a half-angular acceptance of, *e.g.*, ±12°, the measured value of β is reduced by 5% compared to the initial one. This is well corroborated by the PAD simulations described below; changing the analyzer acceptance angle in the SESSA simulation (see below) leads to a reduction of simulated

*Corresponding authors: dupuy@fhi.mpg.de ; bluhm@fhi.mpg.de

β values in agreement with Eq. S2. However, the stated angular acceptance of commercial analyzers is only in the non-dispersive direction. Angular acceptance in the dispersive direction is most likely smaller and quite different, and depends on the analyzer settings (*e.g.*, the entrance slits). Thus, the actual effect of angular acceptance would also have to be determined empirically.

For the same reasons mentioned above, we ignore the angular-acceptance effect here. In principle, the empirical determination of total depolarization effects (angular acceptance plus depolarization of the beam) would be possible by, *e.g.*, measuring the PAD of an s orbital of a rare gas, for which β = 2 at all photoelectron kinetic energies. In practice, because of the combination of these two different effects, depolarization is likely to depend on both the photon energy (beam depolarization) and on the electron kinetic energy (analyzer settings), which makes it little practical to determine. The measurement of β = 2 at high eKE for gas-phase H_2O in key reference 1 suggests that depolarization effects are small, at least in this specific study.

2 Analytical approximation of the DCS

In key reference 1, the DCS was approximated by a Gaussian:

$$\mathrm{DCS}(\theta) \propto \mathrm{e}^{\frac{-\theta^2}{2\phi^2}} \tag{S3}$$

with a characteristic angle $\phi = 17°$ extrapolated from gas-phase water data. For a Gaussian DCS, it is possible to calculate analytically $\mathrm{I}^*(\theta)$ from Eq. 2 in the main text and to identify the modified anisotropy β^*:

$$\beta^* = \beta \frac{\mathrm{e}^{-2n\phi^2}}{1 + \frac{\beta}{4}(1 - \mathrm{e}^{-2n\phi^2})} \tag{S4}$$

Despite its simplicity, this analytical model is relatively robust since Eq. S4 turns out to be valid for non-Gaussian DCS. Calculating the n-fold convolution of a nascent PAD with various DCS and deriving the resulting value of β yields results that can be effectively fitted with Eq. S4, with ϕ as a free parameter. This is shown in the supplementary information of key reference 3.

For $n \to 0$, the expression reduces to:

$$R_\beta = \frac{\beta^*}{\beta} = 1 - \left(1 + \frac{\beta}{4}\right) 2n\phi^2 \tag{S5}$$

3 SESSA simulations

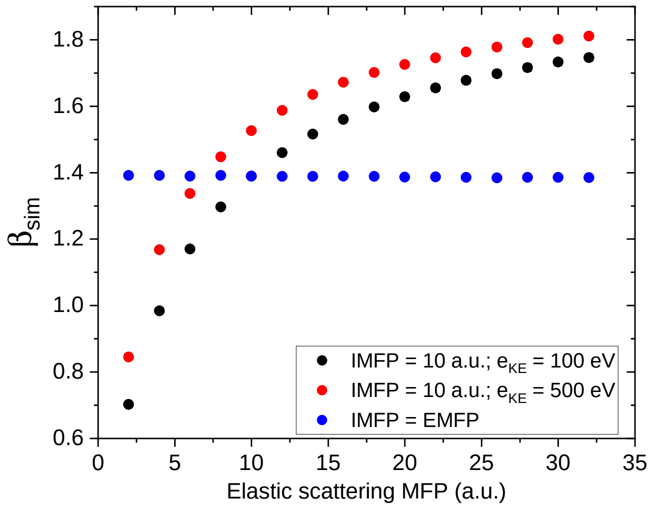

Figure S1: Simulated β values of O 1s photoelectrons as a function of EMFP in SESSA [2] simulations of water. The IMFP and eKE were changed as indicated in the legend. MFP values are arbitrary units.

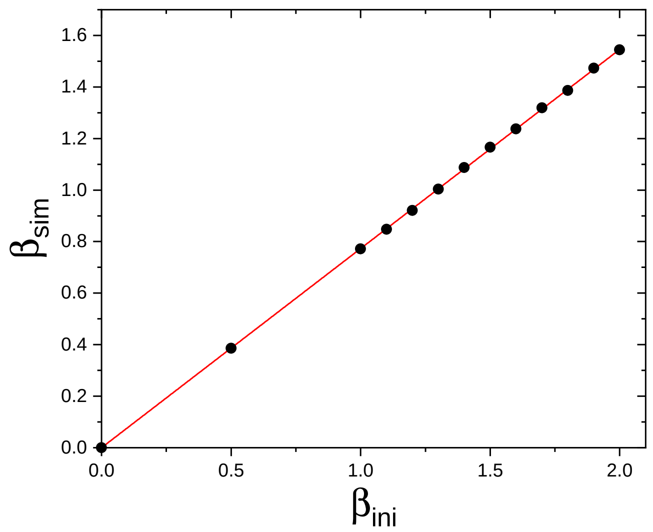

Figure S2: Simulated β values, β_{sim}, for O 1s photoelectrons from a solid water model (see SI), as a function of the nascent β_{ini}. The eKE was set to 100 eV, EMFP = 8 a.u. (arbitrary units) and IMFP = 12 a.u.

SESSA allows to perform simple simulations that provide an intuitive understanding of how the measured β parameter varies as a function of several parameters, especially the IMFP and EMFP. In Fig. S1, we simulated a solid water model (modeled by a homogeneous 2:1 H:O solid sample) and calculated the β value as a function of EMFP, also explicitly varying the IMFP and eKE. The initial β was always 2. The blue points correspond to calculations where we set EMFP = IMFP, *i.e.*, a constant elastic-to-inelastic ratio. This means a constant average number of collisions for the photoelectrons, leading as expected to a constant value of β, independent of the absolute value of the EMFP. EMFP = IMFP is a situation close to what would be roughly expected for water for eKE ∼100-150 eV and higher, which in turn does lead to a constant R_β [3]. The red and black points in Fig. S1 correspond to calculations where we set IMFP = 10 (a.u.) and varied the value of EMFP, effectively varying the EMFP-to-IMFP ratio. We observe a characteristic curve shape, reminiscent of the one obtained in Fig. 1 of the main text, but which, however, should not be confused with it, since when eKE is varied also the IMFP and DCS will change. The red and black points correspond to eKE = 100 eV (black) and 500 eV (red), respectively, the only difference between the two eKEs being the shape of the DCS (since EMFP and IMFP are explicitly fixed), with the DCS at 100 eV containing a significant backscattering component, thus enhancing β reduction. Simulations in a cylindrical geometry, performed in the manner of Ref. [1], showed no significant difference with the plane geometry.

In Fig. S2, we simulated PADs and calculated their β parameters for different values of the nascent β, which is another parameter that can be freely changed in the software. The results show a perfectly linear correlation.

References

[1] Giorgia Olivieri, Krista M. Parry, Cedric J. Powell, Douglas J. Tobias, and Matthew A. Brown. Simulated photoelectron intensities at the aqueous solution–air interface for flat and cylindrical (microjet) geometries. *Phys. Chem. Chem. Phys.*, 19(9):6330–6333, 2017.

[2] Werner Smekal, Wolfgang S. M. Werner, and Cedric J. Powell. Simulation of electron spectra for surface analysis (SESSA): a novel software tool for quantitative Auger-electron spectroscopy and X-ray photoelectron spectroscopy. *Surf. Interface Anal.*, 37(11):1059–1067, November 2005.

[3] Stephan Thürmer, Robert Seidel, Manfred Faubel, Wolfgang Eberhardt, John C. Hemminger, Stephen E. Bradforth, and Bernd Winter. Photoelectron Angular Distributions from Liquid Water: Effects of Electron Scattering. *Phys. Rev. Lett.*, 111(17):173005, October 2013.
